# The unusual case of dyspnea: a pancreaticopleural fistula

**DOI:** 10.1002/ccr3.1434

**Published:** 2018-04-10

**Authors:** Shashank Singh, Mikhail Yakubov, Mukul Arya

**Affiliations:** ^1^ Department of Medicine New York‐Presbyterian Brooklyn Methodist Hospital Brooklyn New York 11215; ^2^ Department of Gastroenterology New York‐Presbyterian Brooklyn Methodist Hospital Brooklyn New York 11215

**Keywords:** Chronic pancreatitis, dyspnea, pancreaticopleural fistula, pleural effusions

## Abstract

Dyspnea secondary to a pleural effusion is a common presentation all clinicians observe, however, usually leads to anchoring the diagnosis to cardiopulmonary etiologies. The formulation of a differential diagnosis encompassing the history of a patient cannot be emphasized enough and is paramount for accurate diagnosis, as described in this case.

## Introduction

Acute and chronic pancreatitis is relatively common disease processes that are well explained in medical literature; however, the rare complication of the development of a pancreaticopleural fistula (PPF) is infrequently described. These patients initially present with respiratory complaints, hence the diagnosis may at first be attributed to a cardiopulmonary etiology. Clinicians must therefore have a high index of suspicion to appropriately diagnose and treat these patients. We present a case of a PPF that developed in a patient with chronic pancreatitis secondary to acute alcoholic pancreatitis, which was diagnosed and managed to resolution. We focus on clinical presentation, diagnosis, and management of these fistulas.

Pancreatic pleural fistulas are a rare condition resulting in an abnormal connection between the pancreas and pleural space. Often associated with recurrent pancreatic damage/trauma leading to chronic pancreatitis, these are a type of internal fistula that results in a chronic and often recurrent pleural effusion, requiring accurate diagnosis and correction 1. The incidence of any pancreatic fistula formation ranges from 10 to 20% depending on the etiology (i.e. iatrogenic procedure complication vs pancreatic trauma) 2, 3. The initial presentation of shortness of breath may divert clinicians toward a cardiopulmonary etiology, leading to inadequate treatment and persistence of the fistula. Persistence of PPFs can lead to infection causing significant morbidity and mortality. We present a case of a PPF and describe its presentation, diagnosis, and treatment.

## Case Presentation

**Figure 1:**
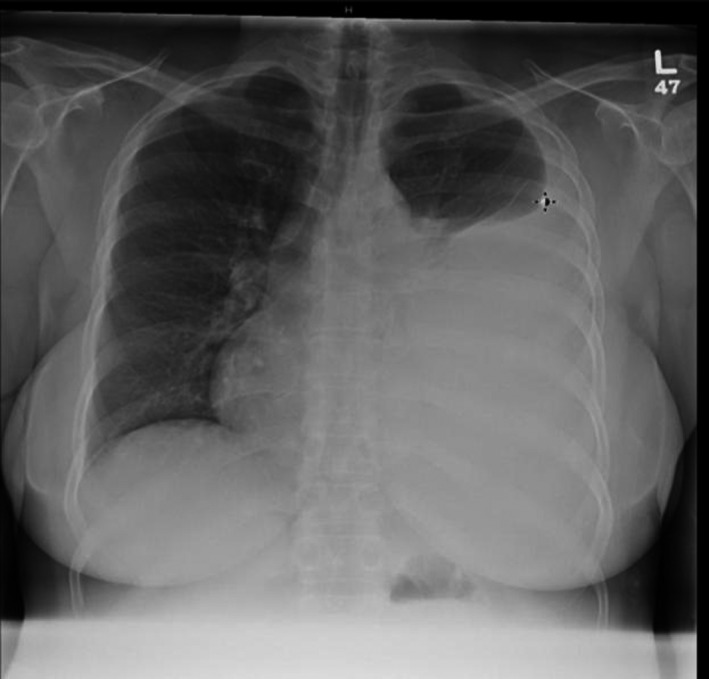
Initial chest x‐ray on ED presentation with large left pleural effusion; evidence of right‐sided tracheal deviation.

**Figure 2:**
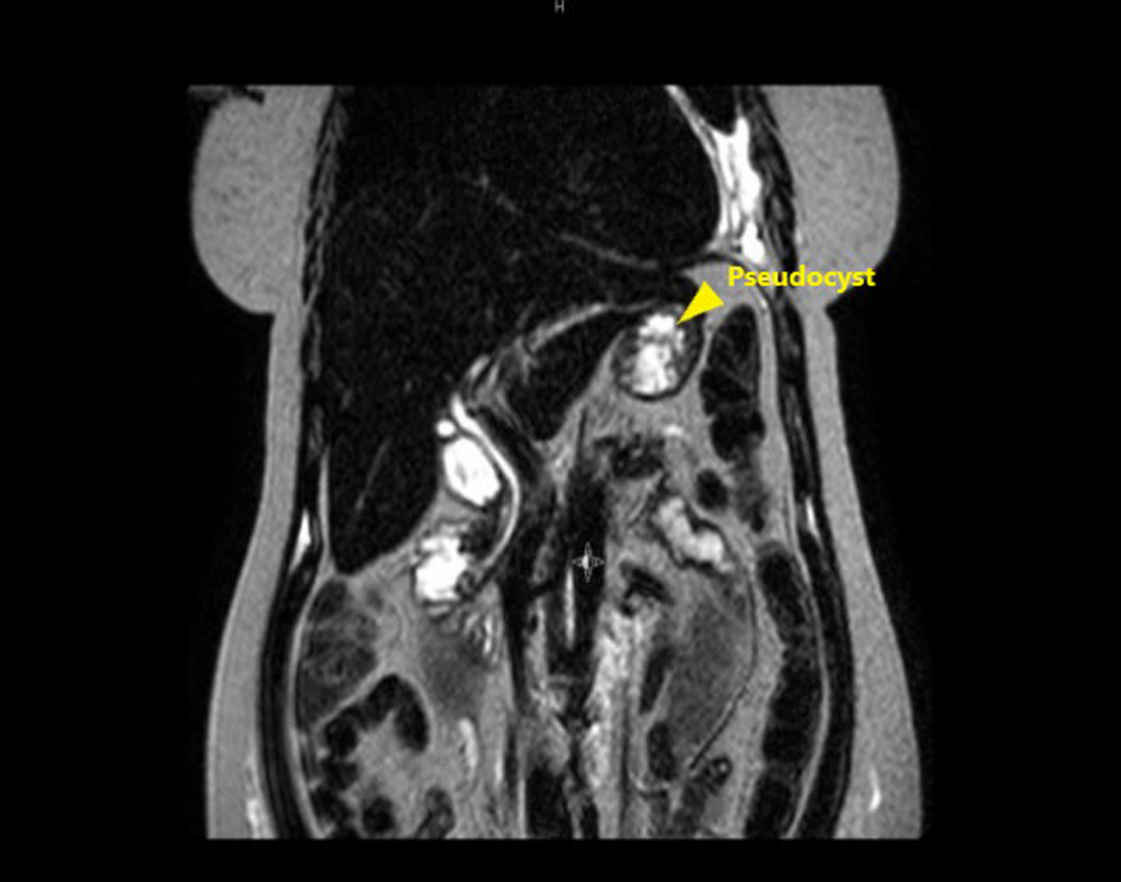
MRCP with evidence of acute pancreatitis and distal pancreatic pseudocyst.

**Figure 3:**
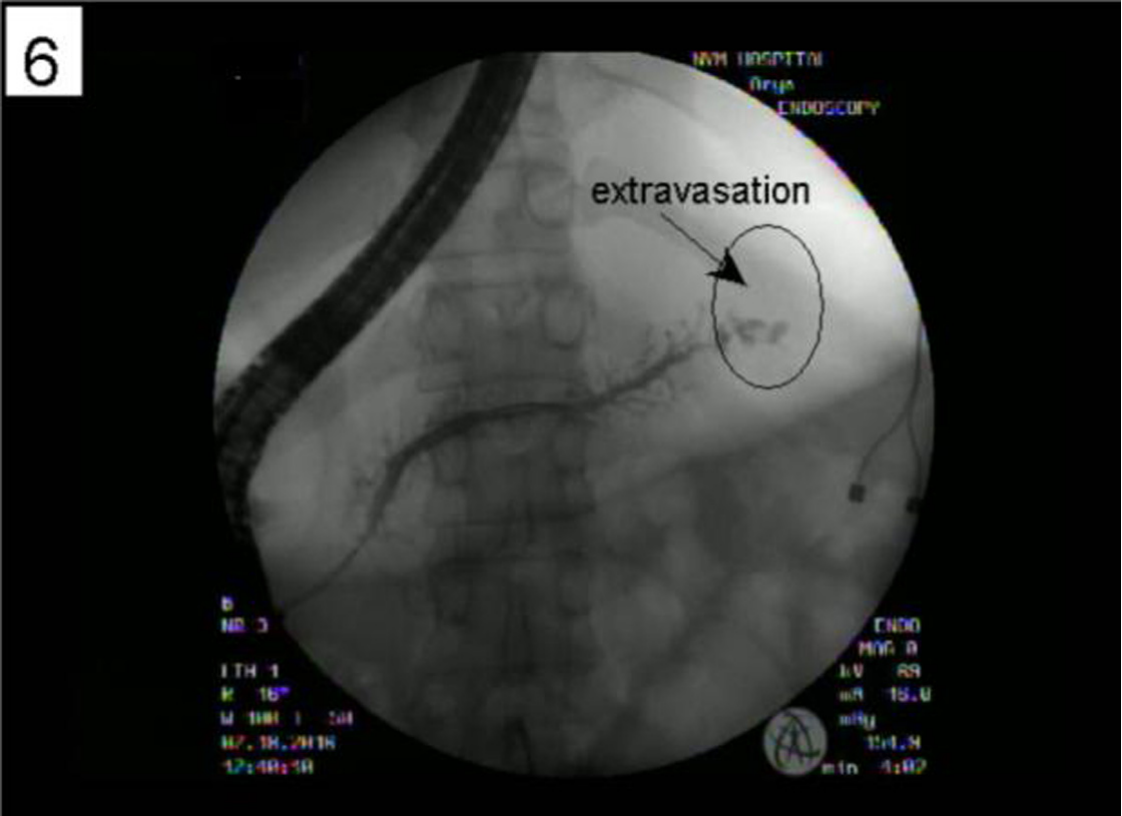
ERCP with evidence of distal pancreatic extravasation of contrast material.

A 46‐year old woman with a history of chronic pancreatitis secondary to recurrent acute pancreatitis from chronic alcohol abuse presented to an urgent care center with complaints of progressively worsening shortness of breath for 2 months, exacerbated with exertion. She was found to have a large opacification of the left lung field and was sent to our emergency room. Initial chest X‐ray (Fig. 1) showed a large left pleural effusion. She underwent a left lung thoracentesis showing an exudative effusion with an amylase level >6500 unit/L; there was no evidence of infection or malignancy. She continued to have rapid accumulation of her left pleural effusion over the next 24–36 h and a pigtail chest tube catheter was placed. Magnetic resonance cholangiopancreatography (MRCP) (Fig. 2) revealed a distal acute pancreatitis complicated by a pancreatic pseudocyst. Secondary to the patient's rapid re‐accumulation and continued chest tube output, medical management was deferred and an endoscopic approach was favored. She underwent an endoscopic retrograde cholangiopancreatography (ERCP) showing an extravasation of contrast mixed with methylene blue in the tail of the pancreas (Fig. 3), and a pancreatic duct stent was placed in the tail of the pancreas. Thereafter, the patient had decreased chest tube output with sustained resolution of her symptoms allowing her chest tube to be removed. She was discharged on hospitalization day sixteen and continued to have appropriate outpatient follow‐up. A repeat ERCP performed 3 months later showed a resolution of her fistula leading to subsequent removal of her pancreatic stent.

## Discussion

This case illustrates an example of a PPF, a rare complication of chronic pancreatitis which can be difficult to diagnose if not considered. Secondary to a pleural effusion, the initial presentation of PPFs in about 65% of cases is the symptoms of shortness of breath, 27% of cases was cough, and 23% of cases was chest pain 4. This can lead one into thinking of a cardiopulmonary etiology, but abdominal pain maybe prominent and thoracentesis will show an exudative effusion that is amylase rich 4, 5. These effusions have a high likelihood of recurrence 5. Once suspected, imaging to visualize the tract is performed. Several imaging modalities can be used including computed tomography (CT), MRCP, and ERCP which have varying sensitivities. MRCP is most sensitive at 80%, followed by ERCP (78%) and CT (47%) 6. Initial imaging with MRCP is preferred as it is noninvasive with a relative high sensitivity and can be helpful in dictating further management 6, 7.

Management can be broken down into a noninvasive and invasive therapy, however, with the advent of ERCP, operative surgical therapy has become a second line treatment, although still definitive 6, 8. Initial medical management consists of thoracentesis, keeping the patient NPO, and somatostatin analogs to decrease pancreatic secretions to allow the fistula track to close 6, 9. Conservative medical management is reported to be successful, and a review of the literature suggests 2–3 weeks of conservative management prior to further therapy 2, 6, 9. During this time, the patient is further managed with parenteral nutrition with continued clinical assessment for fistula closure 10. If unsuccessful, an ERCP with stent placement is performed to reduce ductal pressure to allow physiologic drainage, diverting drainage away through the fistula to allow closure 1. One study found that endoscopic therapy alone was successful in 96.4% of patients with a complete resolution over 5 weeks 11. Patients with pancreatic stent placements are followed with diagnostic ERCP performed every 6 weeks to evaluate for fistula closure and stent removal 8. If a stent is unsuccessful, therapy with surgical intervention can be pursued. Surgical interventions largely consist of internal drainage procedures, for example a fistulojejunostomy or pancreaticojejunostomy, with the former carrying a higher recurrence rate at ~35% or complete surgical resection of the fistula 2.

## Conclusion

PPFs remain to be a rare, but significant problem in patients with pancreatic disease. Dyspnea is a symptom that clinicians encounter often and most place any cardiopulmonary etiologies at the top of the differential. However, it is always important to create a differential diagnosis in the context of a patient's history as well, rather than relying on their symptoms alone. A PPF diagnosis requires a high index of suspicion as patients present largely with respiratory symptoms. Analysis of amylase in pleural fluid is paramount for making a diagnosis. In our case, we tested pleural fluid amylase levels due to the patient's history of recurrent acute pancreatitis. The high amylase content led to further imaging studies allowing us to diagnose a PPF. She was initially treated with conservative medical management, however, secondary to the rapid re‐accumulation of her pleural effusion, she underwent an ERCP with pancreatic stent placement leading to a closure and resolution of the PPF.

## Authorship

SS: primary author. All authors had an equal role in the contribution and editing of this case report.

## Conflict of Interest

The authors declare that there is no conflict of interest regarding the publication of this manuscript.
